# 158. Hepatitis B Screening Among Persons With HIV Receiving Care from the U.S. Department of Veterans Affairs

**DOI:** 10.1093/ofid/ofae631.044

**Published:** 2025-01-29

**Authors:** Stephen Stone, Rachel R Lee, Lauren Beste, Elliott Lowy, David Ross, Amy Weintrob, Rachel V Denyer

**Affiliations:** Washington D.C. VA Medical Center/George Washington University, Washington, District of Columbia; Washington D.C. VA Medical Center/George Washington University, Washington, District of Columbia; VA Puget Sound Health Care System, Seattle, Washington; VA Puget Sound, Seattle, Washington; Office of Specialty Care Services, Veterans Health Administration, Washington D.C., District of Columbia; Washington DC VA Medical Center/ George Washington University, Washington, District of Columbia; Washington DC VA Medical Center/ George Washington University, Washington, District of Columbia

## Abstract

**Background:**

In the United States, around 8% of persons with HIV (PWH) are coinfected with Hepatitis B (HBV), influencing antiretroviral choice and conferring higher mortality. Despite recommendations for universal HBV screening in PWH, small US and international studies found 52-68% were ever screened. National US data on HBV screening in PWH that could inform quality improvement efforts is lacking.

Hepatitis B Screening in Persons with HIV Receiving Care in the US Veterans Health Administration

**Figure:**
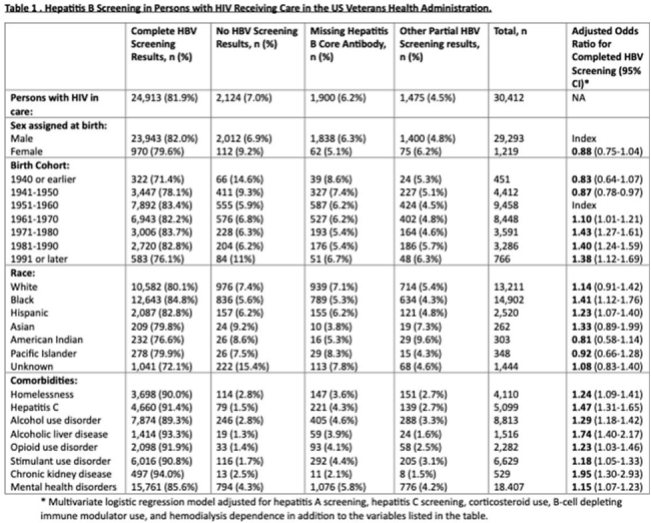

**Methods:**

We used the Veterans Health Administration (VHA) Corporate Data Warehouse to conduct a service evaluation of longitudinal operational data collected during routine care in the integrated electronic health record. We assessed the proportion of PWH receiving HIV care through VHA (at least one encounter in the current or previous year) who have complete HBV serologic screening, defined as at least one result for each of hepatitis B surface antigen (sAg), core antibody (cAb) and surface antibody (sAb). We conducted two multivariate logistic regressions on complete HBV serologic screening using (1) demographic and comorbidity variables, and (2) geographic and facility variables.

Hepatitis B Screening Amongst Persons with HIV by Geographic and Facility Factors

**Figure:**
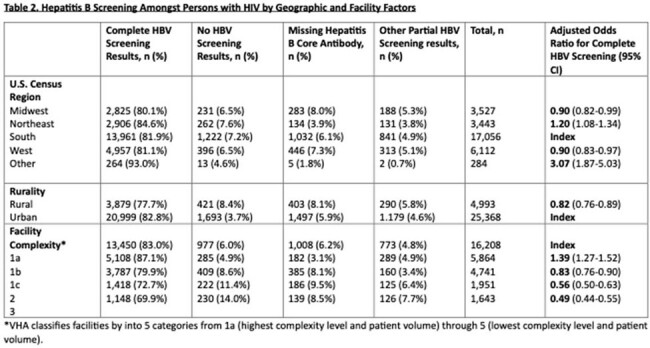

**Results:**

Of 30,412 PWH receiving VHA care, 24,913 (81.9%) had complete HBV serologic screening. Adjusted odds ratios (AOR) for complete HBV screening were significantly higher in PWH who were black or Hispanic, born from 1961 onwards, were homeless, or had comorbidities such as hepatitis C, mental health and substance use disorders, as shown in Table 1, but did not differ significantly by sex. AOR for complete HBV screening were significantly lower for PWH seen at rural and lower complexity (smaller) facilities as shown in Table 2. Among non-immune PWH without hepatitis B (sAg negative, no prior reactive sAg), 55% subsequently received at least 1 dose of hepatitis B vaccine after a median 370-day interval (IQR 75-1512) as shown in Figure 1.

Time to Administration of At Least One Dose Of Hepatitis B Vaccine in Persons with HIV with Negative Hepatitis B Surface Antigen Lacking Prior Positive Surface Antibody Results.

**Figure:**
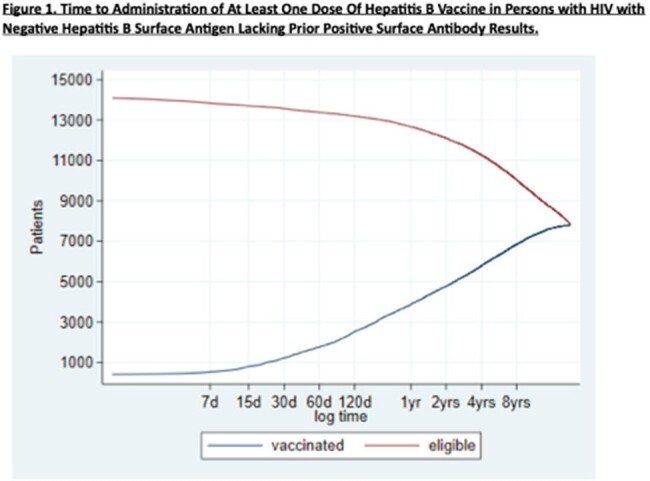

**Conclusion:**

Our data provide insight into current HBV screening practices for PWH in a nationwide US health system. Overall HBV screening rates were high, but 18.1% of PWH had incomplete HBV screening with cAb most frequently missing. Lower odds of HBV screening in smaller facilities and rural populations underscore the need for tailored strategies to ensure equitable access. Our data will inform quality improvement efforts to enhance screening practices for HBV among Veterans living with HIV.

**Disclosures:**

**All Authors**: No reported disclosures

